# UP States Protect Ongoing Cortical Activity from Thalamic Inputs

**DOI:** 10.1371/journal.pone.0003971

**Published:** 2008-12-18

**Authors:** Brendon O. Watson, Jason N. MacLean, Rafael Yuste

**Affiliations:** Howard Hughes Medical Institute, Department of Biological Sciences, Columbia University, New York, New York, United States of America; Porter Neuroscience Research Center, National Institute of Mental Health, United States of America

## Abstract

Cortical neurons in vitro and in vivo fluctuate spontaneously between two stable membrane potentials: a depolarized UP state and a hyperpolarized DOWN state. UP states temporally correspond with multineuronal firing sequences which may be important for information processing. To examine how thalamic inputs interact with ongoing cortical UP state activity, we used calcium imaging and targeted whole-cell recordings of activated neurons in thalamocortical slices of mouse somatosensory cortex. Whereas thalamic stimulation during DOWN states generated multineuronal, synchronized UP states, identical stimulation during UP states had no effect on the subthreshold membrane dynamics of the vast majority of cells or on ongoing multineuronal temporal patterns. Both thalamocortical and corticocortical PSPs were significantly reduced and neuronal input resistance was significantly decreased during cortical UP states – mechanistically consistent with UP state insensitivity. Our results demonstrate that cortical dynamics during UP states are insensitive to thalamic inputs.

## Introduction

Patterned neuronal activations have been postulated to be the potential neuronal scheme for informational representation [1], [2], but they have been relatively difficult to observe and study in the mammalian brain. Recently it has been discovered that such patterned activations arise during UP states, multineuronal depolarizations lasting between 500 milliseconds and 3 seconds in duration. UP states are found in the cerebral cortex both in vitro and in vivo and are particularly prominent during slow wave sleep (SWS) [3], [4], [5]. These depolarized states alternate with hyperpolarized DOWN states, during which neuronal populations are silent. Recently, it has been found that repeatable stable pattern of multineuronal spiking activity occurs with each UP state [3], [6]. Furthermore in slice preparations UP states evoked by thalamic stimulation exhibit the same multineuronal temporal spiking patterns as spontaneous UP states [6]. Work *in vivo* has also demonstrated that these stereotypical patterns of activation arise both during sensory experience and during slow wave sleep [7], [8], [9] suggesting that they are an important operational paradigm of the cerebral cortex.

In this study we set out to evaluate a possible role for UP states. Specifically, we wondered if UP states had a role in information processing or gating: i.e. how cortical networks processes information differently when in an UP state as compared to the DOWN state. On one hand, neurons and networks could be hyper-responsive during UP states, given that depolarization brings neurons closer to threshold for action potential generation, which in turn may allow new inputs to more effectively shape network-wide activity. On the other hand, sensory inputs impinging upon ongoing cortical UP states may not perturb the multicellular firing pattern, since protection of the spatiotemporal pattern would allow functional state to remain intact. In fact, in support of this view, functional imaging experiments in behaving humans demonstrates that ongoing activity tends to suppress newly commanded behavioral output [10]. Simply put, are the UP states and concomitant temporal patterns facilitatory to online dynamical processing of new data? - or do these dynamics carry out a fixed function? [11], [12]. Studies in which single or a few cells are monitored at once could miss the emergent network-level properties of groups of cells. Using thalamocortical slices in combination with imaging of large scale network activity with single cell resolution, we examine how stable network states, characterized by stereotyped multineuronal spatiotemporal dynamics, behave in response to new inputs arriving as they are ongoing. Specifically, we investigate whether the cortical response to thalamic input differs if the cortex is in a DOWN or an UP state. We find that the large majority of individual neurons in layer 4 are insensitive to thalamic input if they are in an UP state and further that multineuronal firing sequences are not perturbed. This insensitivity is likely the result of a major decrease in input resistance that always accompanies the UP state. Our data indicate that UP states render active networks insensitive to thalamic input consistent with the hypothesis that the patterned activity which accompanies the UP state is representative of a specific cortical function.

## Results

### Spontaneous and thalamically-evoked coactivations of groups of layer 4 neurons

**Figure 1:**
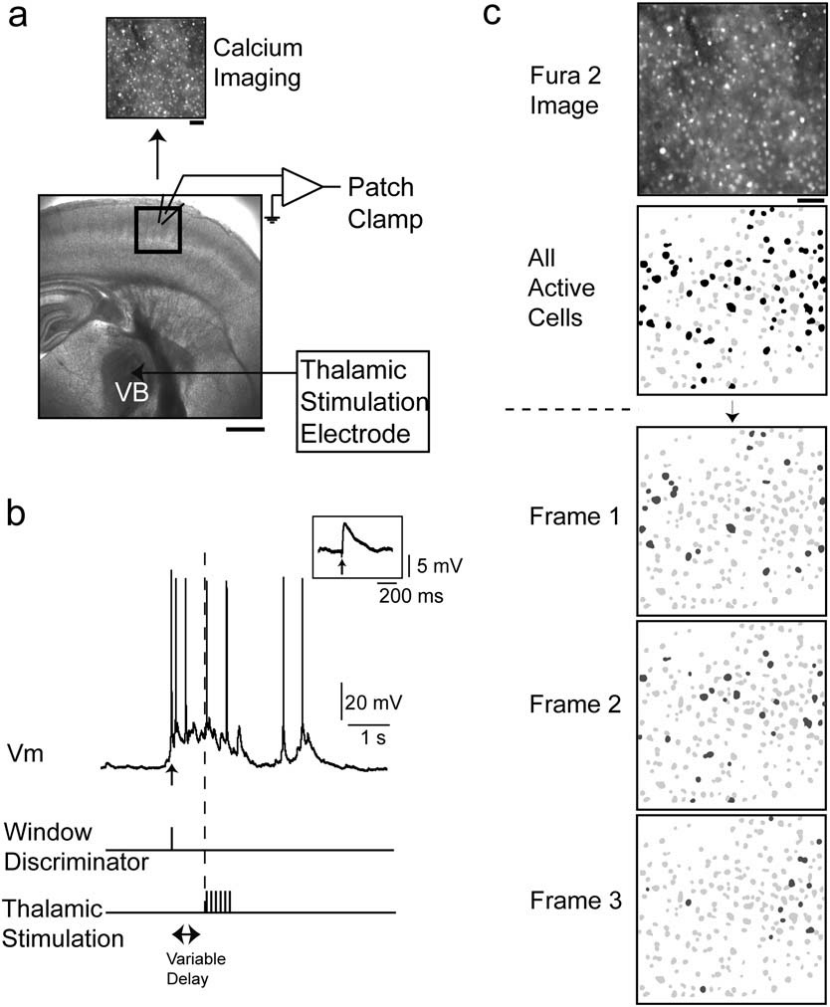
Spontaneous and thalamically-triggered coactivations in thalamocortical slices. (a) Schematic of somatosensory thalamocortical slice preparation. Calcium imaging and whole-cell recordings were made from somatosensory cortex. Bipolar electrical stimulation electrode was placed in the ventral basal nucleus of the thalamus. The region of cortex that responded earliest to thalamic stimulation when imaged at low magnification was chosen for single cell resolution imaging. Scale bars = 1 mm, 50 µm inset. (b). Representative whole-cell recording from a layer 4 neuron revealing spontaneous UP states. This neuron received direct synaptic inputs from the thalamus, as demonstrated by the thalamocortical EPSP observed with every thalamic stimulus (inset). A window discriminator monitored membrane potential in real time and, upon the start of an UP state, activated the thalamic stimulation paradigm from a pulse generator. The delay between the window discriminator output and the initiation of stimulation is under experimental control and was generally set to 350–1000 milliseconds. (c) Representative calcium imaging experiment. Slices were bulk loaded with the calcium indicator Fura 2-AM and network activity was monitored with single-cell resolution by measuring changes in fluorescence. Neurons were recognized automatically and action potential-related activity (spiking cells indicated by filled contours) was used for analysis of spatiotemporal activity patterns. Active neurons were targeted for patch clamp recordings. Scale bar 50 µm.

To investigate the role of cortical UP state activity in the processing of thalamic inputs, we used somatosensory thalamocortical slices from P13-18 mice. This preparation allowed us to stimulate small areas of the ventral basal nucleus of the thalamus, while monitoring the response of layer 4 at both the multi-neuronal level, using calcium imaging, and at the single cell level, with targeted whole-cell recordings (Figure 1). We imaged slices loaded with fura-2 AM to reconstruct, with single-cell resolution, the spiking activity of populations of hundred neurons simultaneously [13], taking advantage of the strict correspondence between calcium transients from fura-2 AM loaded cells and action potentials [14], [15]. We imaged between 142 and 524 neurons per experiment, detecting activated cells with online analysis of movies and then patch clamped 195 of them, allowing for whole-cell recordings and *post-hoc* anatomical analysis. Multiple neurons were often simultaneously recorded.

To activate neurons in layer 4, we used trains of thalamic stimuli (4–8 stimuli, 40 Hz, 25–100 µA) (Figure 1a and 1b), which, in this preparation, generates reverberant cortical activity (Figure 1c), in contrast to low frequency stimulation (<10 Hz), which activates very few cells [16]. This reverberant activity was monitored using calcium imaging and, for the rest of this manuscript, we used the term coactivations to describe groups of cortical neurons that are active coincident with intracellularly recorded UP states. Furthermore, while there was some trial-to-trial variability, the same thalamic stimulation protocol, when repeated, activated highly significantly overlapping populations of neurons within the field of view. Specifically, pairs of thalamic stimuli activated populations of cortical neurons that on average shared 60.2±10.5% of cells (n = 165 movie pairs). This percentage was significantly greater than those found in randomized datasets where active cell identities were reshuffled for each movie (p<0.001 after 10,000 reshuffles; see Methods). In addition, when comparing neuronal calcium transients in pairs of movies, we found that neurons fired in the same sequence from one coactivation to the next to a much greater extent than expected by chance., (see Methods)[6]. On average 67.5±13.6% of active neurons were active in the same temporal sequence across pairs of thalamically triggered cortical coactivations (n = 165 movie pairs; p<0.001 after 10,000 reshuffles of inter-spike intervals (ISI)).

In the same populations of cortical neurons imaged in layer 4, we occasionally detected spontaneous coactivations of neurons, as described previously [6], [17]. These patterns of spontaneous activity also repeated, with pairs of spontaneous activations sharing on average 64.2±14.8% of neurons (n = 69 movie pairs), a percentage much greater that those found in randomized datasets (p<0.001, 10,000 reshuffles). In addition, 60.3±18.7% of active neurons were active in the same sequence across pairs of spontaneous coactivations (n = 69 movie pairs; p<0.001 after 10,000 ISI reshuffles).

Thus, as with thalamic-evoked activity, spontaneous cortical coactivations overlapped significantly. In fact, overall, the populations of neurons activated by thalamic stimulation, and the order in which they were activated, are statistically indistinguishable from those activated in spontaneous synchronizations, as we demonstrated in previous studies [6], [17],

### UP states generated during spontaneous and thalamically-evoked activity patterns

In every neuron that displayed calcium transients during these imaging experiments, patch clamp recordings revealed that thalamic stimulation generated UP states in these neurons, characterized by a long duration depolarization (9.5±3.3 mV amplitude and 2.3±1.2 sec duration, n = 133 neurons; all measures given as mean±standard deviation). These UP states occurred at the same time in simultaneously recorded neurons and were normally accompanied by action potentials (1.1±1.5 AP/sec), demonstrating that intracellular UP states reflect network synchronizations [3], [6], [8], [17], [18]. In these same neurons, spontaneous UP states were observed during long-duration recordings. As previously reported [6], spontaneous UP states were indistinguishable from thalamically-evoked UP states (9.1±3.3 mV amplitude, 2.4±1.2 sec duration and 1.0±1.4 AP/sec, n = 71 neurons; all differences with p>0.1 by t-test), and they also occurred in neurons recorded simultaneously.

We were interested in the roles of neurons receiving direct thalamic input as we felt these neurons would be particularly informative in regards to the effect of UP state activity on impinging inputs. Of 195 neurons recorded from, 19 received direct monosynaptic input from the thalamus, characterized by a reliable, short latency EPSPs after every thalamic stimulation pulse (Figure 1b inset; latencies: 5.2±1.0 ms from stimulus onset, ranges 3.9 to 7.6 msec; amplitudes: 8.2±5.7 mV, ranging from 1.6 to 20.6 mV) [6], [16], [17], [19].

We also recorded by chance from four pairs of monosynaptically connected cortical cells. Their respective monosynaptic potentials were depolarizing with a mean amplitudes of 0.58±0.50 mV (n = 160 trials), 1.02±0.50 mV (n = 160 trials), 1.81±0.86 mV (n = 94 trials) and 2.11±1.03 mV (n = 79 trials) respectively. Latencies were determined from averaged traces and were 1.35 ms, 1.42 ms, 2.12 ms and 1.89 ms for these four cells respectively.

### All major classes of layer 4 neurons participate in cortical UP state coactivations

**Figure 2:**
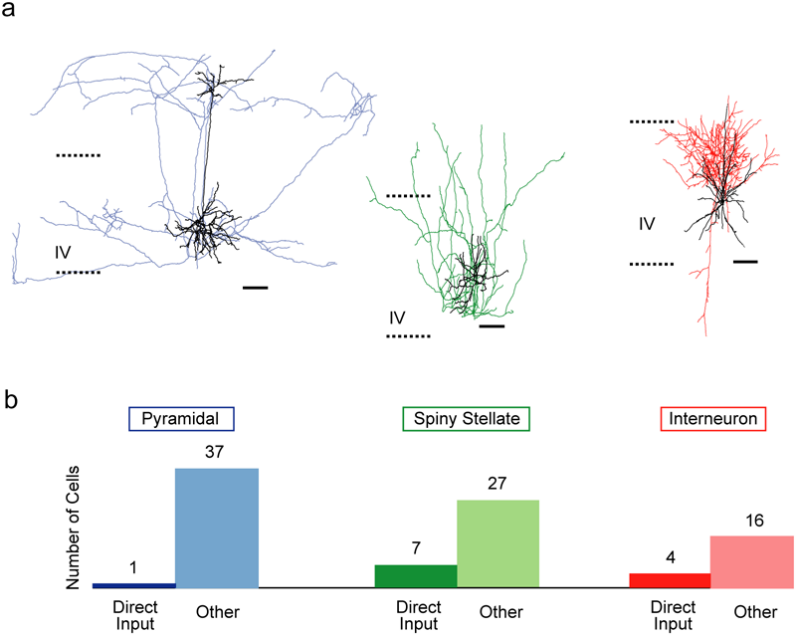
All major classes of neurons participate in cortical UP states. Morphological reconstructions of biocytin-filled neurons indicates that all 3 main classes of neurons in the barrel field were patch clamped and participated in UP state network activations. (a) Morphological reconstructions with dendrites in black, axons in color. Pyramidal cells (left, blue), spiny stellate cells (middle, green) and interneurons (right, red) all demonstrated spontaneous or thalamically-evoked UP states. Layer 4 boundaries indicated with dashed lines. Scale bars 50 µm. (b) Classification of each type of cell observed in our sample of 92 reconstructed neurons that had UP states. Bar graph also shows each class broken down into neurons which did (left bars of each color) or did not (right bars) demonstrate direct input from thalamus (as evidenced by EPSPs with every thalamic stimulation).

As a further tool in our investigation of how thalamic inputs interact with ongoing cortical UP states, we felt it necessary to assess the activity of the various cortical cell classes during these events. To the lay the groundwork for this analysis, we characterized the types of neurons participating in UP states and examined whether participation in these UP state synchronizations was limited to particular anatomical classes of neurons. We therefore set out to determine the identity of the neurons that were activated by UP states. Cells were characterized based on the pattern of action potentials generated upon somatic current injection and also filled with biocytin, allowing for *post hoc* identification of their morphological characteristics. We found that all major classes of layer 4 neurons participated in UP state activations (criteria detailed in the Methods section below), and of 92 morphologically recovered cells, 38 were pyramidal neurons, 34 were spiny stellate cells, and 20 were interneurons (Figure 2). Neurons were classified utilizing the Petilla nomenclature [20]. Of the 103 cells whose morphologies were not recovered, 93 had continuous and sometimes adapting firing patterns, similar to those found in identified pyramidal or spiny stellate cells, whereas 10 had fast action potential kinetics with large sharp afterhyperpolarizations, like those found in some identified interneurons. Therefore the combined anatomical and physiological data indicate that all classes of neurons in layer 4 participate in the cortical UP state coactivations.

In addition, we explored the correspondence between direct thalamic input and cell class identity. We found that a significant proportion of the spiny stellate cells (7/34) and interneurons (4/20) received direct thalamic inputs and only 1 out of 38 pyramidal neurons did (Figure 2b). Although this was not the object of our study, these data indicative a potential bias in the functional effect of thalamic inputs towards spiny stellate cells and interneurons, versus pyramidal cells.

### Lack of effect of thalamic stimuli on ongoing cortical coactivations

We first examined whether the coactivations present during either spontaneous or thalamically evoked cortical UP states could be perturbed by additional thalamic inputs. The stimulation applied to the thalamus during cortical UP states was identical to the stimulation protocol capable of driving the thalamus to initiate cortical UP states when the cortex was in a DOWN state. To deliver thalamic stimuli during spontaneous UP states, we used a window discriminator to monitor the membrane potential of patch clamped neurons participating in UP states (Figure 1b). This window discriminator detected the onset of an UP state and then triggered a train of thalamic stimuli. We also triggered secondary thalamic stimulations during the course of thalamically triggered UP states. In both cases we controlled the delays (which ranged from 6.3 ms to 3.2 sec) between the beginning of the UP state and the impinging thalamic stimulation. Recordings from neurons receiving monosynaptic inputs from the thalamus demonstrated that, in this experimental protocol, the thalamus was indeed activated by stimulations delivered during both spontaneous UP states and thalamically triggered UP states (Figure S4).

**Figure 3:**
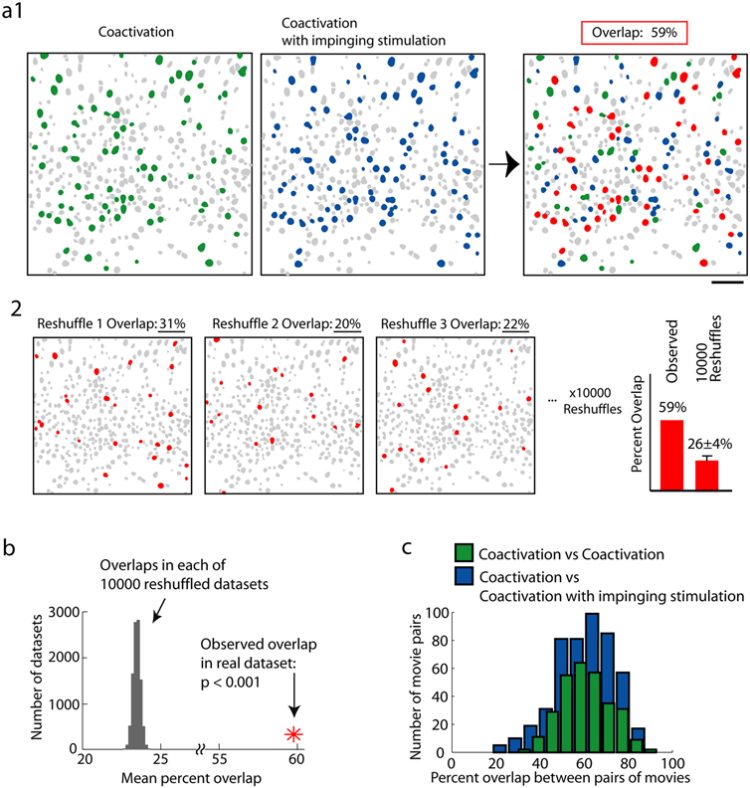
Cortical coactivations are not affected by thalamic stimulation. Patterns of neurons activated consistently in UP states are similar, regardless of additional thalamic inputs to cortex. (a1) Comparison of all active cells imaged during a thalamically triggered coactivation (in green, at left) and during a thalamically triggered coactivation during which the thalamus was stimulated a second time (in blue, at center). Inactive neurons are gray and active neurons are colored. Right: Neurons activated in both movies (“Overlap” cells comprising 59% of the possible overlap) are shown as in red. (a2) Representative overlaps between randomized versions of the movies shown in a1 in which the identities of active neurons was shuffled, to determine what percent overlap should be expected by chance. Right: comparison between observed and expected overlap between this pair of movies (different with p<0.001). Scale bar 50 µm. (b) Mean overlap of dataset of all pairs of movies with and without impinging inputs (red star) versus overlaps calculated from reshuffled datasets (gray histogram of means from populations). The observed overlap across the population was significantly greater than expected by chance (p<0.001). (c) Overlap between all pooled cortical coactivations having no impinging inputs and those with impinging thalamic stimulation (blue) was identical to that between pairs of coactivations without impinging inputs (green). The difference between the means of these distributions was not significant (p>0.10).

To monitor the effect of thalamic stimulation on cortical UP states, we compared and contrasted the patterns of cellular activity during coactivations without impinging inputs (here called “C”) and coactivations with impinging inputs (“CI”) (Figure 3a). We also broke down our data into coactivations initiated spontaneously without impinging input (“sC”), coactivations initiated spontaneously with impinging input (“sCI”), coactivations triggered by the thalamus without additional impinging input (“tC”) and coactivations triggered by the thalamus with an additional impinging input (“tCI”).

Remarkably, while stimulation of sufficient frequency applied to the thalamus during the DOWN state reliably triggered cortical neuronal coactivations, an identical thalamic stimulation during a spontaneous UP state did not appear to have any effect.

In our quantitative analysis, we first tested whether the number of neurons which became active between these two conditions was different, by assessing whether the additional input to the cortical circuit recruited more (or less) neurons than if the cortical coactivation were unperturbed by thalamic stimulation. Overall, in movies of cortical coactivations (C), 100±31.6% of the average number of neurons were active from event to event (n = 88 movies), while in movies where an impinging thalamic stimulation was delivered (CI), 96.0±28.2% of the number of neurons in movies of C type activations within the same population were still active (n = 65 movies). This difference was not statistically significant, as tested by 10,000 bootstrap resamplings of the dataset (p>0.10). Movies of spontaneous coactivations with impinging inputs (sCI) (101.1±35.1%; n = 28 movies) did not show any significant difference in the number of activated neurons when compared with movies with spontaneous coactivations without impinging input (sC) (100±31.6%, p>0.10). Similarly, movies of thalamically triggered coactivations with impinging inputs (tCI) (102.7±28.4%; n = 36 movies) did not show more or less cells than movies of thalamically triggered coactivations without impinging input (tC) (100±23.4%, p>0.1; n = 43). These results demonstrated that thalamic stimulation did not significantly change the number of activated neurons in either spontaneous or thalamically-evoked coactivations.

We then assessed whether the identity of activated cells present during cortical coactivations was the same following impinging thalamic input. Although the total number of neurons was similar in both conditions, the exact population of activated cells could still be different. We tested this by analyzing the overlap of the population of activated cells in consecutive experiments in cortical UP states and those receiving impinging thalamic inputs. In the following analyses of coactivation overlap (and later sequence sharing), we systematically asked the following questions: 1) is there significant overlap between pairs of CI movies, despite the impinging input, 2) is the overlap between CI and C movies greater than what would be expected by chance and 3) is the pattern preservation between C and CI not just significant, but in fact identical to what would occur without any stimulation at all. These three questions were addressed in datasets of a) pooled spontaneous and thalamically triggered coactivations (C, CI) b) spontaneous coactivations alone and (sC, sCI) c) thalamically triggered coactivations alone (tC, tCI).

In the dataset of all pooled spontaneous coactivations and thalamically triggered coactivations, the overlap observed in CI versus CI comparisons was greater than would be predicted by chance, based on 10,000 reshuffles of which neurons were active in each movie, demonstrating the existence of repeated patterns of cell activation in these coactivations with thalamic input impinging upon them (mean = 60.9±11.4%; n = 160 movie pairs; reshuffled mean = 24.2±3.0%; different with p<0.001). In addition the overlap between cells active in C versus CI comparisons was also far greater than in reshuffled datasets, indicating that the coactivation pattern present in coactivations in general was preserved in coactivations with impinging inputs (mean = 60.2±13.4%; n = 485 movie pairs; reshuffled mean = 23.4±2.4%; different with p<0.001) (Figure 3b). Perhaps most importantly, the overlap in C versus CI comparisons was not different than the overlap in C versus C comparisons, indicating that pattern repeatability was the same, regardless of impinging inputs during coactivations (mean = 60.9±11.4%; n = 295 movie pairs; not different with p>0.10 by 10 000 means of re-sampled distributions; Figure 3c). In other words, the same cells were repeatedly activated in the same way despite impinging thalamic inputs.

Separate analysis of spontaneous network UP states and thalamically triggered coactivations also revealed no difference between conditions. Specifically, sCI versus sCI comparisons were much greater than chance (mean = 52.1±13.95%; n = 58 movie pairs; reshuffled mean = 22.8±2.3%; different with p<0.001). Overlaps in comparisons of sC versus sCI were also much greater than expected by chance (mean = 55.9±16.8%; n = 135 movie pairs; reshuffled mean = 21.4±0.6%; different with p<0.001) and were not significantly different from sC versus sC comparisons (64.7±14.8%; n = 67 movie pairs; not different with p>0.10 by resampling; Figure S1a).

Similarly, cellular patterns in thalamically triggered coactivations were not perturbed by thalamic input impinging during the coactivation. Overlap in tCI versus tCI movie comparisons were greater than expected by chance (mean = 67.6±10.0%; n = 90 movie pairs; reshuffled mean = 25.7±1.9%; different with p<0.001). Overlaps in tC versus tCI comparisons were also highly significantly greater than chance (mean = 65.0±10.0%; n = 240 movie pairs; reshuffled mean = 25.0±0.3%; different with p<0.001) and were not significantly different from tC versus tC comparisons (60.2±10.5%; n = 165 movie pairs; not different with p>0.10 by resampling) (Figure S1b).

Thus, across all UP states tested, not only were the patterns of activated cells statistically similar with or without additional thalamic input, but they were also similar across conditions. These analyses ruled out the possibility that impinging thalamic stimuli during a cortical UP states recruits a different or additional population of neurons from those active spontaneously or following thalamic input during the DOWN state.

### Multineuronal temporal sequences of activity are not perturbed by thalamic inputs

**Figure 4:**
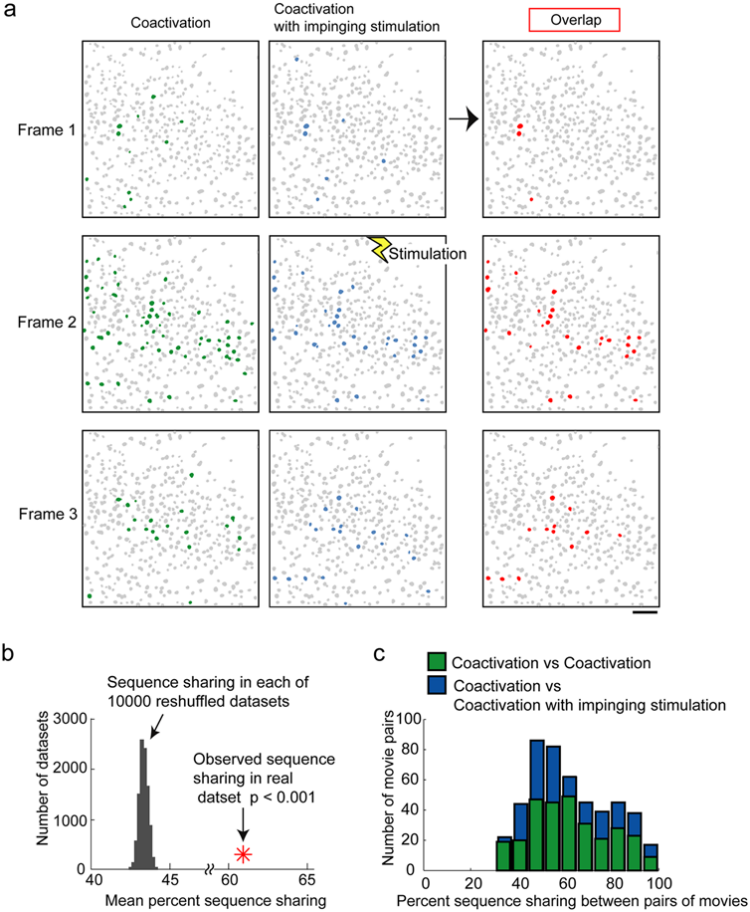
Temporal activation patterns are not altered by thalamic stimulation. (a1) Sequential frame-by-frame activity imaged during a thalamically triggered coactivation (in green, at left) and during a thalamically triggered coactivation during which the thalamus was stimulated again (in blue, at center). Inter-frame interval was 300 ms. Right: Neurons activated in the same sequence in both of these movies in red. A one frame jitter was allowed between any pair of movies, but in only one constant direction. Scale bar 50 µm (b) Mean sequence sharing from dataset of all pairs of movies with and without impinging inputs (red star) versus from reshuffled datasets (gray histogram of means from populations). All sequence sharing calculated as percent of neurons all overlapping between a pair of movies that are active in the same sequence. The observed sequence sharing across the population was significantly greater than expected by chance (p<0.001). (c) Sequence sharing between movies of cortical coactivations having no impinging inputs and those with impinging thalamic stimulation (blue) was identical to that between pairs of coactivations without impinging inputs (green). The difference between the means of these distributions was not significant (p>0.10).

In addition to the identity of coactive neurons being the same from coactivation to coactivation, it has been established that neurons fire in the same temporal sequence [6], [8]. Although we were unable to detect any change in which neurons were activated during cortical coactivations when thalamic inputs arrived, we wondered whether the characteristic sequential activation of neurons that occurs during cortical coactivations could be perturbed by a thalamic stimulation, impinging while the sequence was progressing. Indeed, if the specific sequence of activation of neurons carries information[7], it is conceivable that the specific role of thalamic inputs could be to alter these temporal sequences. We tested this by analyzing sequence sharing in a dataset of pooled spontaneous and thalamically triggered coactivations (Figure 4a). First, we find that sequence sharing in CI versus CI comparisons was greater than that in 10,000 randomized datasets where spike train interspike intervals were reshuffled, demonstrating the existence of a temporal code in these coactivations (mean = 64.1±16.9%; n = 157 movie pairs; reshuffled mean = 43.7±0.5%; different with p<0.001). The amount of shared sequential activation in C versus CI comparisons was also much greater than in reshuffled datasets revealing that the multicellular firing sequences present in coactivations without impinging input are also present in the coactivations receiving impinging inputs (62.6±16.8%; n = 480 movie pairs reshuffled mean: 45.6±0.3%, different with p<0.001) (Figure 4b). We also found that this sequence sharing in the C versus CI comparisons above was not merely present but was in fact not different than that in C versus C comparisons providing evidence that the sequences are not interrupted or changed by new inputs (mean = 62.2±16.7%; n = 292 movie pairs; not different with p>0.10 after 10,000 bootstrap resamplings of the dataset) (Figure 4c).

Again, we found no difference when this analysis was performed on either spontaneous or thalamically triggered coactivations alone. Sequence sharing was observed at levels much greater than expected by chance in sCI versus sCI comparisons (mean = 64.1±16.9%; n = 58 movie pairs; reshuffled mean = 46.5±0.2%; different with p<0.001). Also, sequence sharing in sC versus sCI comparisons was both greater than in reshuffled datasets (mean = 60.9±20.2%; n = 135 movie pairs; reshuffled mean = 49.6±0.7%; different with p<0.001) and was not different from in sC versus sC comparisons (60.7±18.8%, n = 67 movie pairs; not different with p>0.10 by bootstrap resampling) (Figure S2a).

Similarly, sequence sharing was observed at levels much greater than expected by chance in tCI versus tCI comparisons (mean = 65.6±16.1%; n = 90 movie pairs; reshuffled mean = 48.5±0.4%; different with p<0.001). Sequence sharing in tC versus tCI comparisons was both greater than in reshuffled datasets (mean = 66.4±15.0%; n = 240 movie pairs; reshuffled mean = 44.6±0.3%; different with p<0.001) and was not different from in tC versus tC comparisons (67.5±13.6%, n = 165 movie pairs; not different with p>0.10 by bootstrap resampling) (Figure S2b).

Thus, both the cellular and temporal activation patterns present during cortical coactivations are preserved, regardless of additional thalamic input.

### Lack of effect of thalamic stimuli on action potential generation during intracellularly recorded UP states

**Figure 5:**
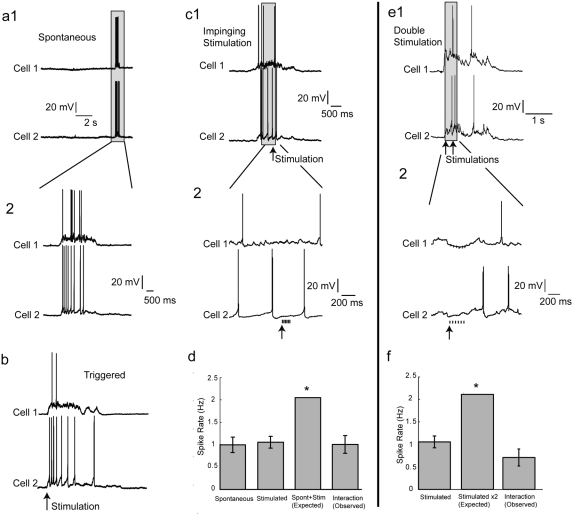
Thalamic stimulation does not perturb membrane potential during UP states. (a) Simultaneous whole-cell recordings in two neurons reveal spontaneously arising UP states. Boxed areas shown at higher temporal resolution below. (b) Simultaneous whole cell recordings of the same neurons reveal UP states following thalamic stimulation that was manually triggered during a cortical DOWN state. (c) Simultaneous whole cell recordings of the same neurons during a spontaneous UP state with an automatically-triggered thalamic stimulation. Arrow indicates time of thalamic activation. Boxed area around the stimulus itself is shown at higher resolution below. There is no observable change in the UP state as a result of the thalamic stimulation occurring during the ongoing UP state. (d) Quantification of population spike rates during spontaneous UP states versus UP states resulting from thalamic stimulation during the DOWN state versus spontaneous UP states with impinging thalamic input. Bars represent means of mean spike rates for each cell, and error bars represent standard error of the mean. None of the measured spike rate means of different types of UP states were significantly different (p>0.10 by bootstrap resampling). The third bar represents a linear summation between spiking during spontaneous UP states and that triggered by thalamic stimulation during the DOWN state. The value calculated in this summation differs significantly from all measured values (by bootstrap resampling, p<.001, indicated by *), including the value of spontaneous UP states with interacting added thalamic stimulation. (e1) Similar recordings of a different pair of neurons during a thalamically stimulated UP state with a subsequent thalamic stimulation occurring during the UP state. Arrows indicate times of thalamic activation. Similar to ongoing spontaneous UP states, there is no observable change in membrane potential as a result of the thalamic stimulation. (f) Quantification of population spike rates during thalamically stimulated UP states versus thalamically stimulated UP states interacting with thalamic input. Bars represent means of mean spike rates for each cell, and error bars represent standard error of the mean. A second thalamic stimulation during an UP state did not increase spiking during UP states (p>0.10). The value calculated in this summation differs significantly from measured values (by bootstrap resampling, p<.001, indicated by *), including the value of stimulated UP states with interacting added thalamic stimulation.

While we were able to demonstrate that there was no effect of impinging thalamic stimulation on the population spiking patterns during UP states, we wanted to use a more sensitive method than calcium imaging to assess the effects on single neurons. We therefore patch clamped 57 neurons and recorded their firing in UP states while we stimulated the thalamus (Figure 5).

Across all neurons in which both spontaneous UP states and spontaneous UP states with impinging thalamic stimulation were recorded (n = 30), all quantified UP state characteristics were statistically indistinguishable between the two conditions: mean amplitude (9.1±3.3 mV for spontaneous vs. 7.8±2.8 mV for spontaneous with input), mean duration (2.4±1.2 s for spontaneous vs. 2.7±1.2 s for spontaneous with input), and average firing rate (1.0±1.4 AP/second for spontaneous vs. 1.0±1.3 AP/second for spontaneous with input) were not different (p>0.1, in all cases by bootstrap resampling; see Methods). In fact, the rate of spiking observed after thalamic stimulation during spontaneous UP states was half of that which would be expected by summation of the spiking from each condition separately (Figure 5d). This implies that the network was saturated during the ongoing spontaneous UP state. More rigorous analysis, using within-cell differences of the mean for each parameter across these two conditions, confirmed that the spontaneous UP state and the spontaneous UP state with thalamic stimulation were not statistically different (differences always spontaneous-with-input minus spontaneous): within-cell differences of mean amplitude across conditions were 0.4±1.6 mV (not greater than zero p>0.10 by t-test), within-cell differences of UP state mean durations were 0.2±0.8 s (not greater than zero p>0.10 by t-test) and within-cell differences of mean spike rates were −0.0±0.9 AP/second (not greater than zero p>0.10).

We then inquired whether there was any effect of thalamic stimulation during ongoing thalamically-triggered cortical UP states. Similar to the results obtained with ongoing spontaneous UP states, we found that there was no prolongation, nor increases in amplitude or spike rate, during stimulation of ongoing thalamically-triggered UP states (Figure 5e and 5f). Across 17 neurons, UP state amplitudes (9.5±3.3 mV for stimulated versus 11.5±4.0 mV for stimulated plus input), durations (2.3±1.2 seconds for stimulated versus 3.3±0.9 seconds for stimulated plus input) and firing rates (1.1 AP/second for stimulated) did not have significantly different means (all p values greater than 0.1 by bootstrap reshuffling). Within cell differences across classes of activations were also not greater than zero indicating the expected facilitatory effect of additional thalamic stimulation during ongoing UP states was missing (triggered–with-input minus triggered): amplitude differences were 0.1±1.0 mV (p>0.10), duration differences were −0.1±0.5 seconds (p>0.10) and firing rate differences were −0.2±0.3 AP/second (p>0.10).

Finally, as expected, due to the similarity of spontaneous UP states to thalamically- triggered ones, the spontaneous UP states with thalamic stimulation were not statistically different from thalamically-triggered UP states in 33 cells in which both types of UP states recorded (Figure 5d). The within-cell difference of mean amplitudes in spontaneous UP states minus thalamically triggered ones were −0.7±2.9 mV (p>0.10), the within-cell differences of UP state mean durations across conditions were 0.05±0.8 s (p>0.10) and the within-cell differences of mean action potentials number per UP state were −0.2±2.0 APs (p>0.10).

We conclude that the activation of thalamic inputs has no detectable effects on large-scale spiking activity during cortical UP states.

### Temporal analysis of the effect of thalamic stimuli on action potential generation

We were surprised by the overall lack of effect of thalamic stimulation during cortical UP states, given that exactly the same stimuli impinging during cortical DOWN state had such a pronounced effect (Figures 5b and 5e1). Since our initial analysis compared all action potentials generated during the UP state, we wondered whether the effect of thalamic stimulation during ongoing UP states could have a temporal signature that could be missed by our analysis. For example, the effect of thalamic stimulation could be a brief and more subtle increase in spiking, only present immediately after the stimulation. This idea is related to the possibility that the neural code could be based on differences in spike timing, rather than one based on average spike rates across long intervals of time [21].

We explored the temporal dependency of the effect of thalamic stimulation on cortical UP states by generating peri-stimulus time histograms of action potentials generated during ongoing UP states, centered on the time of the thalamic stimulus onset (Figure S3). Firstly, we found that the efficacy of the thalamic input in generating a spiking response during UP state activity did not depend on the time of stimulation relative to the start of the UP state. The spike frequency in the immediate post-stimulus time was insensitive to impinging thalamic inputs throughout the duration of the UP state (tested delays from 6.3 ms to 3.2 sec from onset) (Figure S3a).

Additionally, we failed to detect the increase in spiking that would be predicted from summation of the DOWN state post-stimulation spiking response with the baseline UP state firing rate (Figure S3b). The post-stimulus first time bin had a lower than expected amplitude during spontaneous UP states (n = 42 neurons, p>0.10, p values generated by bootstrap resampling), thalamic-evoked UP states (n = 17 neurons, p>0.10) and in the pooled data from both conditions (n = 57 neurons, p>0.10). All responses were similarly found to be less than expected across different bin sizes, or when evaluating the second bin after the stimulus (all p values>0.10). Moreover, not only was the amplitude of the instantaneous response (first or second time bin) less than expected, but there was not a clear prolongation of the response. Furthermore, while the average cortical response to thalamic stimulation during the DOWN state lasted for 2.3±1.2 seconds, even the non-significant increase in spiking during the UP state lasted only 200 ms (significantly lesser duration p>0.10).

Finally, we carried out an evaluation of the effect of thalamic stimulation on the spiking of each individual neuron across all time bin sizes (Figure S3c). We calculated the p value for each cell having greater than chance spiking with each bin size by reshuffling of stimulation times and took the within-cell mean of these p values across bin sizes. Cell p values distributed uniformly, as 34 of 57 neurons showed post-stimulus spiking either less than or equal to that expected from their overall spike rate (p = 1). The remaining 27 cells had p values distributed between 0 and <1 indicating that our observed lack of effect was not due to our measures.

### Analysis of individual cell responses

Although our analysis indicated an overall general lack of effect of thalamic stimulation on cortical activity, we explored more precisely its potential effect in the immediate post-stimulus firing of each individual recorded neuron. For each cell we first calculated the likelihood of seeing the observed number of post-stimulus spikes at any particular time bin, given the general UP state spike rate of that cell. Because of the variability in spiking for each cell for any given trial, we used Monte Carlo simulations to generate a valid null hypothesis. Specifically, we calculated spike rates per bin by simulating 10,000 randomly chosen stimulus times during each UP state and then estimating their p values by calculating how frequently the observed post-stimulus spiking of each neuron over multiple trials was observed by chance. Out of the 57 neurons analyzed, three neurons had significantly more action potentials after the stimulus than could be explained by their ongoing firing rate before the thalamic stimulus across all binning sizes (Figure S4; p<0.05). These three neurons increased their firing to thalamic inputs, when they were in an UP state, by 1300% (n = 2 trials, p = 0.028 over 10 000 reshuffles with 100 ms bins), 680% (n = 3 trials, p = 0.003 over 10,000 reshuffles with 100 ms bins) and 420% (n = 6 trials, p = 0.000 over 10,000 reshuffles with 100 ms bins), respectively. Two of the neurons were part of the population of neurons tested for responses to thalamic input during spontaneous UP states (n = 42 total), whereas the third cell was part of the population tested for responses to inputs during thalamically-triggered UP states (n = 17 total). Inspection of the individual traces revealed that this enhanced spiking was restricted to the time spanning the thalamic stimulation itself (Figure S4c), and was not prolonged as was the effect of thalamic stimulation during the DOWN state.

Because each of these three neurons demonstrated a statistically significant action potential response to thalamic stimulation during UP states, we sought to further analyze their potential contribution to the overall population response. For this purpose, we analyzed whether the increase in the population spike rate in the first or second bin after thalamic stimulation was significantly greater than could be due to chance fluctuations in the baseline rate of the population by using a bootstrap randomization to reshuffle stimulation times. We found that when our entire population of 57 neurons was analyzed, the significance of the increase in firing rate was inconsistent across binning widths (threshold of p = 0.05) - on the edge of statistical significance (Figure S3b and S3d). On the other hand, when we removed these three responding neurons from the population, the remaining 54 neurons showed no significant response at any bin size (p>0.10 at all bin sizes) (Figure S3d).

We then sought to identify whether the three responding neurons had common morphological or physiological characteristics. Physiologically, all three neurons were recipients of direct thalamic inputs (Figure S4b). In fact, within the population of 8 neurons receiving direct thalamic EPSPs evaluated in the interaction experiments, these three cells received particularly large thalamocortical EPSPs (10.3 mV, 12 mV and 20.6 mV on average; population range 3.4–20.6 mV). The remaining 5 direct-input cells did not show, individually or as a group, any significant spiking response to thalamic input (for all cells: p = 0.5 by Wilcoxon). All three responding cells were recovered for anatomical processing and reconstructed. Two were spiny stellate cells, whereas the third was an interneuron, with fast spiking characteristics (Figure S4a).

Despite the responsiveness of these cells, non-direct input neurons recorded simultaneously with them, exhibited no effect in response to thalamic stimulation on the same trials (Figure S4d). This indicates that the increase spiking response was restricted to these three individual neurons, which received unusually large direct thalamic EPSPs, and was not propagated to other portions of the circuit.

### Synaptic inputs in excitatory cells are smaller during UP states

**Figure 6:**
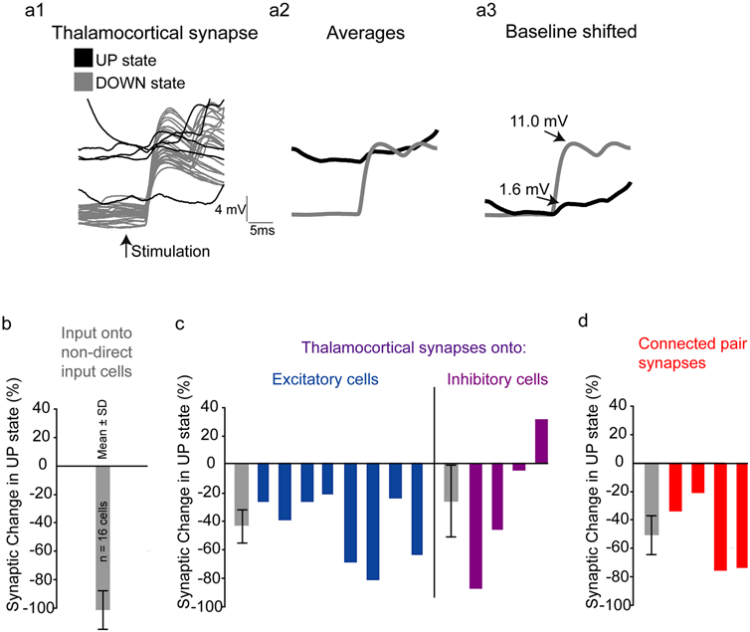
Synaptic efficacy is reduced during UP states. (a) Synaptic inputs are smaller in UP states than in DOWN state. A1: Individual recordings of thalamically-evoked monosynaptic inputs to an excitatory cortical neuron in the baseline DOWN state (in gray) versus in the UP state (in black). A2: Average post-synaptic responses for each condition. A3: Relative amplitude after correcting baseline shift. A clear reduction (85%) is visible in the UP state. (b) Efficacy of synaptic inputs to “downstream” cortical neurons, i.e. those not receiving direct input from the thalamus. Maximal depolarization in a 100 ms window following thalamic stimulation was measured in the DOWN state and at the same time point during the UP state. All measures were relative to a baseline defined by the mean membrane potential for 100 ms before stimulation. Recordings with action potentials occurring during this time were excluded from the analysis. Every neuron examined (n = 16) showed a decrease in depolarization from baseline (population mean was a 103.3% decrease, standard deviation 14.1%). (c) Thalamocortical EPSPs decrease in 8 of 8 excitatory neurons in the UP state compared to the DOWN state. In blue is shown the EPSP percent change in the UP state versus the DOWN state for excitatory cells. Eight out of eight excitatory cells demonstrated smaller EPSPs in the UP state (range of decreases: 21%–85%, mean: 43.4%, standard deviation 23.3%; see gray bar at left of graph). In purple are EPSP percent changes for 4 recorded interneurons, which show heterogeneous changes during the UP state ranging from a 30% increase to a 65% decrease (mean: 26.5% decrease, standard deviation 50.0%; see gray bar at left of graph). (d) Decreases in EPSP amplitudes between four monosynaptically connected pairs of cortical neurons (range of decreases: 20.0–73.5%; mean 49.6%, standard deviation: 27.1%; see gray bar at left of graph). All postsynaptic cells were excitatory in these pairs.

Our results demonstrated a generalized cortical insensitivity to thalamic input during UP states, something which was surprising in its extent. To better understand the mechanisms responsible for this phenomenon, we examined the effect of UP states on the synaptic inputs received by cortical neurons after thalamic stimulation. We first analyzed “downstream” neurons, which did not receive monosynaptic inputs from the thalamus (and whose synaptic inputs must therefore presumably originate from the cortex), comparing the average depolarization caused by thalamic inputs during DOWN and UP states. Sixteen cells were chosen for this analysis and all of them demonstrated a decrease in the average depolarization at 100 ms after thalamic stimulation during cortical UP states relative to the depolarization during DOWN states (mean decrease of 103.3±14.1% SD; p<0.05 by Wilcoxon; Figure 6b). We then assessed this relative depolarization at all time points between 0 and 100 ms and obtained similar results, with all 16 neurons demonstrating reduced depolarization (80.4±42.5%, range 21.7–156% decrease; p<0.05 by Wilcoxon). Thus, “downstream” neurons received a smaller excitatory drive after thalamic stimulation during UP states than during DOWN states.

We then examined the effects of UP states on thalamocortical EPSPs in neurons receiving them directly (Figure 6a). In 8 out of 8 excitatory cells examined, we measured a reduction in average EPSP peak amplitude in the UP state, as compared to the DOWN state (Figure 6c blue; 43.4% mean reduction of peak amplitude; range from 21.4%–85.5% decrease; p<0.05 by Wilcoxon). Note that even in these cells, we were able to record distinct EPSPs during UP states, indicating that the thalamus was effectively activated and transmitting to cortex. However, a heterogeneous response was observed in interneurons: of four examined, one demonstrated a 65% reduction in EPSP peak amplitude, the second showed a 6% reduction, the third a 4.9% reduction and the fourth cell actually exhibited a 30% increase in the maximum amplitude of its EPSPs during UP states (Figure 6c purple). Furthermore, this fourth interneuron was one of the 3 highly responsive neurons described above (Figure S4, middle panel) and was in fact induced to spike on the first thalamocortical EPSP during each UP state. In contrast, this neuron never fired an action potential during the first EPSP in a train during the DOWN state. Thus, this inhibitory cell was likely to provide inhibitory inputs to neurons downstream more readily and faster when the network was in the UP state, and may in fact be representative of a class of interneurons in the cortex that could have this function. This cell displayed very fast action potential kinetics with sharp after-hyperpolarizations and morphologically was a multipolar interneuron with descending axonal projections.

We also analyzed the average amplitudes of corticocortical synapses from four connected pairs of neurons (all post-synaptic neurons were excitatory) during both UP states and DOWN states. Consistent with our observations of thalamocortical synapses, we found a strong decrease in amplitude of those single-axon corticocortical synapses during UP states, as compared to DOWN states (mean 49.6% decrease, range: 20.0–73.5%; Figure 6, red). This result implies that a generalized mechanism for the reduction in synaptic efficacy exists in cortical circuits during UP states.

The overall reduction in the efficacy of thalamocortical synaptic transmission provides a clear mechanistic counterpart to our phenomenological observation of a lesser cortical response to thalamic stimulation in the UP state than in the DOWN state. Furthermore a subclass of interneurons may exist which could damp the cortical response and may also provide a network-level explanation for what we observe.

### Input resistance in excitatory cells is reduced during UP states

UP states are thought to be characterized by baseline changes in membrane potential and membrane resistance [3], [22], [23]. In fact both of these changes in baseline state occur in the proper direction to potentially explain the observed decreased synaptic efficacy: the depolarization may lead to loss of driving force and the decreased resistance may have a shunting effect. We sought to determine which of these two mechanisms might contribute to the generalized decrease in synaptic efficacy we observe. To test the effect of driving force, we depolarized direct input neurons during the DOWN state to a membrane potential similar to that they experience during UP states (as determined in earlier recordings), and measured the size of thalamic EPSPs at those two potentials. We found that there was no decrease, but in fact a non-significant increase in the amplitude of thalamocortical inputs with depolarization (mean 9.5%, range: 5.1–17.5%) consistent with previous reports [27].

**Figure 7:**
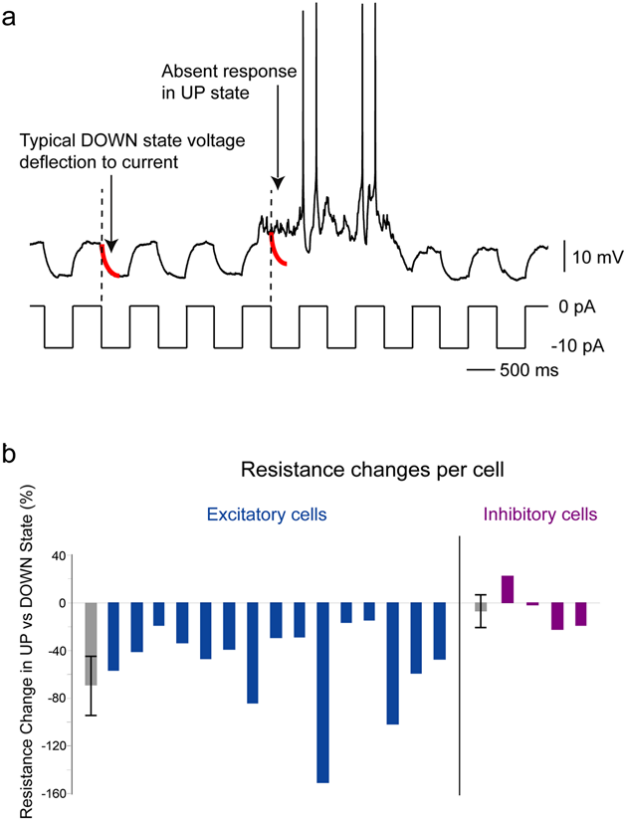
Cellular input resistance is decreased during UP states. (a) Cellular input resistance was assessed in UP states and DOWN states by injecting a constant series of hyperpolarizing pulses. In this example, a typical DOWN state voltage deflection in response to a current pulse is highlighted in red at left. When this is overlaid on the UP state voltage trace at the time of current injection, little or no response is observed. Averaging of the voltage deflections across all recorded DOWN states and all recorded UP states for each neuron allowed us to calculate the input resistance change between these two conditions. (b) 15 of 15 excitatory neurons showed statistically significant decreases in input resistance during UP states compared to DOWN states (blue, at left). Mean decrease was 50.9%; standard deviation was 36.6%, shown in gray bar at left. 2 of 4 interneurons showed significant decreases in resistance during the UP state and one cell actually showed an increase. Mean inhibitory interneuron decrease was 4.9%; standard deviation was 20.2%, shown in gray bar at left.

After ruling out a driving force effect, we focused on testing whether cortical neurons could be shunted during UP states. In order to assess membrane resistance changes during UP states, we injected hyperpolarizing current pulses into recorded neurons during UP and DOWN states (Figure 7). We found that 15 of 15 excitatory neurons (spiny stellate, pyramidal or regular spiking) had statistically significant decreases in input resistance (p<0.05 for each cell after 10,000 reshuffles of UP versus DOWN state identity of each current pulse), with a significant change in population mean (−50.9±36.6% change, range −14.1 to −150.0%; p<0.05 by Wilcoxon) (Figure 7b blue). Also, 2 of 4 neurons with either morphology or spiking patterns consistent with inhibitory neurons showed a significantly decreased resistance in UP states (p<0.05 after reshuffles), 1 out of 4 showed no change in resistance and one showed an increase in resistance during UP states (Figure 7b purple). There was no significant change in the population mean (−4.9±20.2% change, range: −22.3 to 21.8%; p>0.10 by Wilcoxon).

While the interneuron showing a larger thalamocortical EPSP is not the same neuron as that demonstrating increased input resistance during the UP state (not all tests were performed on all neurons), these two cells showed remarkably similar fast spiking patterns with similar action potential and after-hyperpolarization kinetics in response to direct current injection (Figure S5).

These results indicate that the decrease in synaptic amplitude in excitatory neurons during UP states could be explained by the decrease in resistance measured in the same cells. In addition, the variable response of interneurons during UP states also mirrored the variable effects on thalamocortical efficacy in interneurons during UP states.

## Discussion

Using population calcium imaging and targeted patch recordings in thalamocortical slices, we demonstrate that the stereotyped patterns of multineuronal activations present during both spontaneous and thalamically triggered UP states are essentially unaffected by thalamic inputs. Furthermore we provide evidence that the lack of propagation of the thalamic signal through the cortical circuit is likely due to reduced synaptic efficacy caused by decreased cellular input resistance during UP states.

### Insensitivity of cortical neurons to thalamic input during UP states

As indicated by imaged activity, when we delivered thalamic inputs during ongoing cortical activity, we did not observe any difference in either the number or identity of neurons that were activated. In addition, we did not detect a perturbation of the temporal sequences of activation during UP states with impinging stimuli (Figure 4a). This was striking, as stimuli that had been strong enough to trigger full network activations during network quiescence did not alter an ongoing spatiotemporal sequence when delivered during network coactivations. Thus the multineuronal firing patterns during UP states are not interrupted, perturbed or modified by thalamic stimuli.

Using patch clamp recordings from a subset of active neurons we also found that thalamic stimulation during UP states fails to modify ongoing activity, as recorded intracellularly (Figure 5). Again, the lack of effect of the thalamic stimulation is particularly striking if one considers the expected number of action potentials after thalamic stimulation based on DOWN state responses (Figures 5d, 5f and Figure S3b). Our data, taken from mouse somatosensory system *in vitro*, generally agree with the previous single cell results from Petersen *et al.* [24], Sachdev *et al.* [25] and Hasenstaub et al [26] in anesthetized animals. However, in contrast to these past studies, performed at the single cell level, the use of multicellular calcium imaging in conjunction with patch clamp allowed us to assess these questions at the level of entire circuit. Thus, UP states render the majority of cortical neurons and, most importantly, the entire circuit insensitive to incoming thalamic inputs even in layer 4, where the effect of thalamic input would be expected to be greatest (Figure 5).

### Synaptic and cellular mechanisms of cortical insensitivity

Stimulating the thalamus during UP states revealed that thalamocortical EPSPs onto excitatory neurons are significantly reduced, during these states (Figure 6). To assess which aspects of UP states contribute to this decreased synaptic efficacy we mimicked the depolarization which occurs during UP states in cortical neurons but were not able to attribute the decrease in synaptic efficacy to a decrease in driving force. On the contrary, the amplitudes of PSPs slightly increased with intracellular depolarization, consistent with previous findings [27] that demonstrated that voltage-gated conductances in the soma increase the amplitudes of synaptic inputs when neocortical pyramidal cells are depolarized. We then attempted to assess whether the depolarization which defines the UP state exhibits a corresponding decrease in input resistance for cortical neurons. We found a concomitant decrease in input resistance during UP states in 100% of excitatory neurons tested. On the other hand, inhibitory interneurons displayed a more varied change in both EPSP amplitude and input resistance during UP states, which may mirror the varying roles inhibitory cells likely play in circuit function.

Measurements of input resistance or conductance, generally carried out at the soma, may not properly examine their effect on inputs, since most excitatory inputs are located in spines which are electrically isolated from the dendritic shaft [28]. For this reason, we view EPSP measurements as a more direct measure of the receptivity of neurons to incoming inputs. Indeed, using targeted patch clamp recordings of neurons which receive direct input from the thalamus we also detect a major reduction in thalamocortical EPSP size. Our data imply that the local resistance encountered by these synaptic inputs is lower during UP states. These observations are also supported by experiments performed *in vivo* [29].

### Network mechanisms of network insensitivity

Out of 57 neurons examined we found three neurons that had significant responses to thalamic inputs during UP states (Figure S4). All three neurons received direct thalamic input and had EPSPs greater than 10 millivolts in amplitude – which could clearly contribute directly to spiking. Although it is possible that these neurons represent a preferential “labeled line” it is also possible that these large EPSPs could be a consequence of the extracellular stimulation method used, which may enhance synchronization of convergent thalamic axons. This seems likely since single axon thalamocortical EPSPs are estimated to be much smaller than 1 mV [29].

Not only were these three responsive neurons a minority of the direct input cells, but most importantly, none of the ‘downstream’ neurons – those neurons located within layer 4 which did not receive detectable direct input from thalamus - displayed an increase in spiking following stimulation during UP states. Using patch clamp recordings of connected cortical neurons we also found that corticocortical synaptic inputs were depressed during ongoing UP states, indicating a failure of the signal to propagate through the circuit, beyond these few responsive cells (see also Figure S4d). Furthermore, the responses observed in these three above-mentioned cells were restricted to only the time of the stimulation itself. This is in stark contrast to the cortical response to thalamic input during the DOWN state, which typically outlasts the stimulation by seconds. Our data thus clearly demonstrates lesser overall network engagement during UP states.

Mechanistically, it appears that the failure of propagation through the network is the result of the increased conductance during UP states. Indeed, we find that neurons in UP states have half the input resistance they do in DOWN states. How does this 50% reduction lead to a 100% loss of network response to inputs in the circuit? It seems that while the largest of inputs may evoke spiking in a subset of the first layer of recipient cells (i.e. 3 out of 12 direct input neurons), the fact that only a few neurons in layer 4 spike in response to input, combined with the fact that all neurons are in a low input resistance state, means that downstream layers receive smaller inputs while in UP states. This decrease would be summed with each subsequent layer of neurons, until threshold is not reached at all and spiking does not occur. Thus the network-wide change in receptivity during UP states, combined perhaps with the nonlinearity inherent in the action potential threshold, could render the entire active network altered to an extent greater than the change in any one neuron.

### Functional implications

It is interesting to consider what could be the functional role of this cortical insensitivity during UP states. It could be argued, based on our data, that the UP state is a period of ongoing processing which is insensitive to outside input, in analogy to the “Fixed Action Patterns” described by ethologists. Given that the neurons that participate in spontaneous UP states can be activated in quite precise spatiotemporal patterns [3], [6], [8], [17], [18], UP states could in effect protect these sequential dynamics, and possibly corresponding stereotypical behavioral patterns, from impinging sensory inputs.

In a potentially related set of studies, patterns of multineuronal activation have been described during “windows” of action potentials in SWS consistent with UP states [7]. These patterns of multicellular spiking are repetitions of sequences occurring during waking behavior [7], [9] and their replay during SWS may be the signature of network-wide consolidation of the memories of events, especially given that memory consolidation has been shown to occur during SWS. Alternatively these repeating and evocable sequences of activation may be related to a more fundamental and general information representation process which occurs upon activation of a particular area of cortex - representing favored network states which are activated whenever a particular portion of cortex is properly engaged. Therefore, the persistence of these states in neocortical circuits is in good agreement with their putative yet crucial role in neural processes.

In either case, the purpose of the mechanisms responsible for the cortical insensitivity that we observe could be to protect these stable circuit dynamical states, allowing them to function properly and rendering the UP state a special protected functional state. This then is consistent with the postulates of de No and Hebb that multineuronal sequences of activity are crucial to the functioning of the brain [1], [2].

## Materials and Methods

### 

#### Slice preparation

Thalamocortical slices, 400 µm thick, were prepared from postnatal day 13 (P13) to P18 C57BL/6 mice, as previously described [6]. Slices were cut with a vibratome (VT1000S; Leica, Nussloch, Germany or Microm 650V, ThermoFisher Scientific, Kalamazoo, Michigan) in ice-cold oxygenated modified ACSF that included 0.5 mM CaCl_2_ and 3.5 mM MgSO_4_, in which NaCl was replaced by an equimolar concentration of sucrose. Experiments were performed with ACSF containing (in mM) 123 NaCl, 3 KCl, 26 NaHCO_3_, 1 NaH_2_PO_4_, 2 CaCl_2_, 2 MgSO_4_ and 10 dextrose, which was continuously aerated with 95% O_2_, 5% CO_2_. All experiments were performed in the absence of any ionic or pharmacological manipulations but with high perfusion and oxygenation rates.

#### Imaging

Slices were bulk loaded with Fura 2-AM for visualization of action potential-related activity in neuronal somata. Slices were placed onto the bottom of a small Petri dish (35×10 mm) filled with a vortexed mixture of 2 ml ACSF, an aliquot of 50 µg Fura 2-AM (Molecular Probes), 15 µl DMSO and 2 µl Pluronic F-127 (Invitrogen, Carlsbad, CA). A cover was placed over the petri dish and it was incubated in the dark at 35–37°C and oxygenated by puffed CO2/O2 gas for ∼25 minutes.

In order to locate regions in the cortex connected to the area of thalamus we stimulated, we first imaged at low (4×) magnification. The region which responded earliest to stimulation was then chosen for higher cell resolution imaging and patch clamping (Figure 1a).

Changes in intracellular free Ca2+ were visualized with a 20× or 40× Olympus Plan FL objectives with an upright fluorescence microscope (Olympus BX50WI; Olympus Optical, Tokyo, Japan) using a 380 nm excitation filter, a 395 nm dichroic mirror, and a 510 nm emission filter (Chroma Technology, Brattleboro, VT). A Hamamatsu C9100-12 (Bridgewater, NJ) camera and Simple-PCI software (Compix Imaging, Sewickley, PA) were used for all presented imaging data. A Princeton Instruments Micromax (Trenton, NJ) with IPLab software (Scanalytics, BD Biosciences, Rockville, MD) were used for targeting neurons for patch clamp in experiments from which no data was included in the imaging dataset. Frames were acquired at 300 ms/frame and in the case of the Hamamatsu camera, a 6.25% or 1.56% neutral density filter was inserted to decrease the excitation light in order to minimize bleaching. Binning was performed such that images were 256×256 pixels. Files were saved as multipage tiffstacks.

#### Imaging Data Analysis

Detection of action potential-related calcium transients was performed using custom written software as previously [6]. In brief, after high pass filtering of raw images outlines of neuronal cell bodies were detected using a combination of brightness and size thresholds [3]. To analyze activity, framewise percent changes were calculated for each pixel to create images of frame-to-frame changes. A baseline noise level was calculated from the standard deviation of the pixels outside of cells. Neuronal activations were detected from each of these images by recognizing bright areas containing at least a minimum number of contiguous pixels each with brightness more than 2 times the noise level (5 pixels in 40× movies, 8 pixels in 20× movies). Once these were detected, if their center of mass overlapped with the location of a known cell outline, that cell was recorded to be “on” in that frame. If a cell was found to be active in more than one frame in a row, only the first activation was recorded.

For all analyses presented here, only activations occurring during series of contiguous frames corresponding to UP states were included. These frames had to form a contiguous series of at least 500 milliseconds combined duration each containing a number of cellular activations equaling the greater of the following two numbers: three or median cells per frame in the movie plus two (usually the latter).

#### Electrophysiology

Thalamocortical projection neurons were activated using bipolar platinum-iridium electrodes (#CE2C55, Frederick Haer Co., Bowdoinham, ME) placed in the ventrobasal nucleus (VB) of the thalamus. Stimuli were 200 µs in duration, 20–100 µA in amplitude and were applied individually or as a train of 4–8 stimuli, each separated by 25 ms (40 Hz) using a Master 8 pulse generator coupled to a Iso-flex stimulator (AMPI, Jerusalem, Israel). For each slice the minimal pulse amplitude necessary to evoke recurrent activity was used. Recordings were made at either 37°C or at room temperature and results were pooled since no differences were observed between data collected at these two temperatures. Calcium imaging of populations of neurons [13] was used to do online identification of responding cells in layer 4 and these neurons were then targeted for whole-cell recordings. Whole-cell current-clamp recordings using Axoclamp 2B, Axopatch 1D and Multiclamp 700A and B amplifiers (Axon Instruments, Foster City, CA) were made from neurons in layer 4 using 5–9 MΩ micropipettes, filled (in mM): 130 K-methylsulfate, 2 MgCl_2_, 0.6 EGTA, 10 HEPES, 4 ATP-Mg, and 0.3 GTP-Tris, pH 7.2 (290–295 mOsm). To characterize neurons, 500–1000 ms depolarizing DC current injections were given to each cell and resultant action potential firing patterns were analyzed, following the Petilla convention nomenclature [20]. For interaction experiments, membrane voltage for one of the patch-clamped neurons was fed into a window discriminator (121 Window Discriminator, WPI Sarasota, FL), which was set to trigger off a 10 mV depolarization to activate stimulation of thalamus.

#### Morphological processing

Neurons were filled with biocytin by diffusion from the intrapipette solution during recordings, with electrodes containing 0.4 g/100 ml biocytin in addition to the solution described above. At the end of each recording, slices were fixed overnight in 4% paraformaldehyde. Thereafter, slices were rinsed several times in 0.12 M phosphate buffer saline (PB). Slices were then transferred to 30% sucrose in 15 mL of 0.12 M PB for at least 2 hours and as long as one week. Slices were then frozen in an embedding medium. After freezing, slices were rinsed in 0.12 M PB several times. Slices were then incubated in 1% H_2_O_2_ in 0.12 M PB for 30 min under agitation and rinsed in 0.12 M PB once for 15 minutes. After two other washes in 0.02 M KPBS, the slices were incubated overnight under agitation in 1% Avidin-Biotin Complex (ABC Kit Standard, Vector Laboratories) prepared in 0.3% Triton X-100. After three rinses in phosphate buffer, biocytin was revealed by diaminobenzidine. After two final rinses in phosphate buffer, slices were mounted onto slides. The neurons were reconstructed with Neurolucida (Micro Bright Field Inc., USA).

Neurons were classified in part based on their morphologies. Pyramidal neurons were identified based on their characteristic triangular or round somata, spiny dendrites, large single apical dendrites with an apical tufts, multiple basal dendrites, basal-projecting axon initial segments and continuous or adapting steady state action potential firing patterns in response to current injection. Spiny stellate cells had similar firing patterns and axon initial segments as pyramidal cells, but their dendrites, while spiny, were multipolar. In some cases, axons of pyramidal neurons projected to neighboring cortical columns (Figure 2a), but spiny stellate axons were not observed to do this. Inhibitory interneurons were heterogeneous and were characterized by a wide variety of features including aspiny dendrites of many branching configurations, highly variable axonal morphologies, but with initial segments that did not tend to project basally, and action potential firing patterns that included fast-spiking, continuous spiking and stuttering types

#### Electrophysiology Analysis

UP states were detected automatically from whole cell current clamp traces based on fulfillment of the following minimum criteria: at least 500 ms of depolarization of 3 mV or more and at least 3 action potentials during this depolarization. If the neuron did not fire action potentials, a continuous depolarization of 5 mV for a minimum of 500 ms was required. This allowed us to detect all UP states despite the variability of membrane behavior exhibited by different neurons. Simultaneous patch clamp recordings confirmed that these criteria allowed for the reliable detection of network UP state events which occurred simultaneously in simultaneously recorded cells. Further, after automatic detection, all events meeting these requirements were reviewed by the experimenter and could be rejected at that point. Durations and amplitudes for verified UP states were quantified based on automatically detected UP state start times and stop times. Action potentials were detected based on their amplitudes and durations and numbers within detected UP states were quantified.

Thalamic stimulation times were recorded as 5 V impulses on a separate data acquisition channel simultaneously with intracellular recordings. These were detected using a threshold and those which occurred during UP states were gathered and used as time references for peri-stimulus time analyses.

To evaluate differences in means of non-normal distributions (spike rates, amplitudes and durations of UP states) bootstrapping was utilized [30]. N random individual samples were redrawn from the original set of N observations with each sample able to be drawn any number of times (0 times, once or many times), as determined by a random number generator. This generation of surrogate datasets was repeated 10,000 times for each of the pair of distributions to be compared. Means and differences between means were calculated for each of the 10,000 resamplings, and the p value of the observed mean was calculated by determining the proportion of the surrogates with the observed value.

Similarly, to calculate whether each neuron spiked more in response to thalamic stimulation over all trials than would be expected by chance (Figure S3), spiking was measured in the same way in “response to” 10,000 randomly placed surrogate stimulations. Spiking was most often evaluated within the first bin of some binning scheme. The surrogate stimulations were placed between the start of the UP state and the minimum of 1) the end of the UP state or 2) 1 second into the UP state, in order to control for the fact that UP state spike rates decrease over time. Cell p values were calculated as the percent of the surrogate dataset that the observed value was greater than expected.

Calculation of PSP sizes during a particular condition was performed by first averaging the post synaptic response in all trials during which we recorded a presynaptic stimulus (minimum of n = 3 trials for each measure). This allowed us to make a global measure of efficacy at any given synapse. After this averaging, latency was measured as the start of the rise or fall from baseline and amplitude was measured as the maximum deflection from baseline.

For measurements of input resistance, 500 millisecond hyperpolarizing pulses were delivered with 500 milliseconds in between. Current command was recorded on an independent channel during all experiments. This 500 millisecond duration was chosen to allow for at least half of the duration of each pulse to occur during steady state voltage and was chosen based on the observed DOWN state membrane time constant of the highest resistance cells recorded. Analyses only included measurements from cells which did not demonstrate changes in measured total DOWN state resistance over the course of the experiment. The periods of hyperpolarizing current injection overlapped randomly with UP and DOWN states. UP vs. DOWN state beginnings and endings were defined by the bounds of periods of long-duration depolarization and increased synaptic noise (see Figure 7), and whenever possible from simultaneously recorded neurons which did not receive hyperpolarizing test pulses. Each hyperpolarizing pulse was determined to be in either an UP state or a DOWN state if it did not occur during a state transition. Measurements during state transitions were not used. Resistance calculations were made by comparing the mean voltage in the second 50% of each hyperpolarizing pulse (as this was a steady state measure) with mean the second 50% of the non-hyperpolarized periods both before and after that hyperpolarizing pulse. If the non-hyperpolarized period either before or after the hyperpolarizing pulse occurred during an UP/DOWN transition, it was not used and only one period adjacent to the hyperpolarizing pulse was used for comparison to the hyperpolarized timepoint.

## Supporting Information

Figure S1 Effect of thalamic inputs on coactivation overlaps, segregated by type of UP state. (a) Overlap of pairs of movies during pairs of spontaneous UP state events (green) and pairs comprised of one spontaneous UP state and one spontaneous UP state with impinging thalamic stimulation (blue). The difference between the means of these distributions was not significant (p>0.10). (b) Same analysis carried out with pairs of movies during pairs of thalamically triggered UP state events (green) and pairs comprised of one thalamically triggered UP state and one thalamically triggered UP state with impinging (additional) thalamic stimulation (blue). The difference between the means of these distributions was not significant (p>0.10).
(2.28 MB TIF)

Figure S2 Analysis of effect of thalamic input on temporal sequences overlap segregated by type of UP state. (a) Sequence sharing from pairs of movies during pairs of spontaneous UP state events (green) and pairs comprised of one spontaneous UP state and one spontaneous UP state with impinging thalamic stimulation (blue). The difference between the means of these distributions was not significant (p>0.10). (b) Same analysis carried out with pairs of movies during pairs of thalamically triggered UP state events (green) and pairs comprised of one thalamically triggered UP state and one thalamically triggered UP state with impinging (additional) thalamic stimulation (blue). The difference between the means of these distributions was not significant (p>0.10).
(2.15 MB TIF)

Figure S3 Lack of effect of thalamic stimulation on the temporal activity during ongoing UP states. (a) Plot of percent likelihood of action potential generation in response to thalamic stimulation during ongoing UP states over the duration of the UP state (moving average with 1000 ms bins, 250 ms apart). Time of thalamic stimulation relative to UP state start time does not greatly affect the responsivity of the cortex to thalamic input. Time histograms centered at times of thalamic stimulation in the DOWN state versus the UP state in 57 neurons. (b) Expected versus observed response to stimulation during ongoing UP states) shown in peri-stimulus time histogram (PSTH) format (bin width = 100 ms, stimulation time t = 0 represented as vertical red line). Gray bars show linear summation of firing rate during ongoing UP states with that observed after thalamic stimulation in the DOWN state from all 57 cells examined. Blue bars show observed firing before during and after stimulation during UP states. Following the start of stimulation, there is a small increase in spike rate across all cells, which is significantly greater than baseline under some binning regimes but not others. Also it is significantly less than expected (p<0.05 by bootstrap resampling of first and second bins in observed dataset). (c) Histogram of average p values for test of greater than expected post-stimulus spiking for each cell over analyses using multiple bin sizes. P values calculated by reshuffling stimulation times over the duration of UP states to determine spiking expected by chance. P values for neurons were distributed uniformly, as 34 of 57 neurons showed post-stimulus spiking either less than or equal to that expected from their overall spike rate (p = 1). The remaining 27 cells had p values distributed relatively evenly between 0 and <1. Three neurons demonstrated both a mean p value less than 0.5 but also p values less than 0.5 across each and every bin size. (d) In blue bars is the population peri-stimulus time histogram generated after these 3 cells (5%) were removed from the population. These neurons are explored in further detail in Supplemental Figure 4. The response after stimulation is now not significantly different from baseline under any binning regime. For reference, in gray bars is the PSTH from all 57 cells, identical to the blue bars in part (b).
(3.78 MB TIF)

Figure S4 Three direct-input neurons display increased spiking after thalamic stimulation during UP states. Three neurons showing significantly greater than chance spiking after thalamic stimulation during UP states across all time binning strategies. Each neuron is represented in a column; thalamic stimulations were only delivered during spontaneous UP states in the cells in left two columns, while thalamic stimulations were only delivered during ongoing thalamically stimulated UP states in the right column. (a) Reconstructions of each responding neuron. Layer 4 upper and lower boundaries are indicated by dotted lines. Scale bars 100 um. (b) UP states during which thalamus was stimulated, with stimulus times indicated by arrows. Peri-stimulus times are shown in gray boxes and are shown at higher temporal resolution below. (c) Action potentials are clearly triggered during the period of stimulation, but during UP states are restricted to that time. (d) Recordings made simultaneously with those in the third row, but in other neurons, none of which received direct thalamic input. Consistent with our other observations in 46 other neurons not receiving direct thalamic input, even these cells which are recorded simultaneously with consistently responsive up stream cells demonstrate no spiking response following thalamic stimulation.
(2.54 MB TIF)

Figure S5 Inhibitory interneurons with apparently increased responsiveness during UP states. Side by side comparison of interneurons, one of which received direct input from thalamus (a) and received 29.9% larger amplitude thalamocortical EPSPs during UP states than during down states and the other (b) demonstrated 21.76% increased input resistance during UP states. Upper panels: Both neurons were members of the interneuron subtype with fast spiking in response to depolarizing current injection and had strikingly similar action potential and after hyperpolarization kinetics. Lower panels: Biocytin fills of both cells; each was recorded simultaneously with other neurons. Interneurons of interest indicated with red arrows. Scale bars 10 µm.
(4.24 MB TIF)
