# Supplementary figures and images for: UP States Protect Ongoing Cortical Activity from Thalamic Inputs
Source: PLoS One. 2008 Dec 18;3(12):e3971. doi: 10.1371/journal.pone.0003971 (PMC2597736; doi:10.1371/journal.pone.0003971)

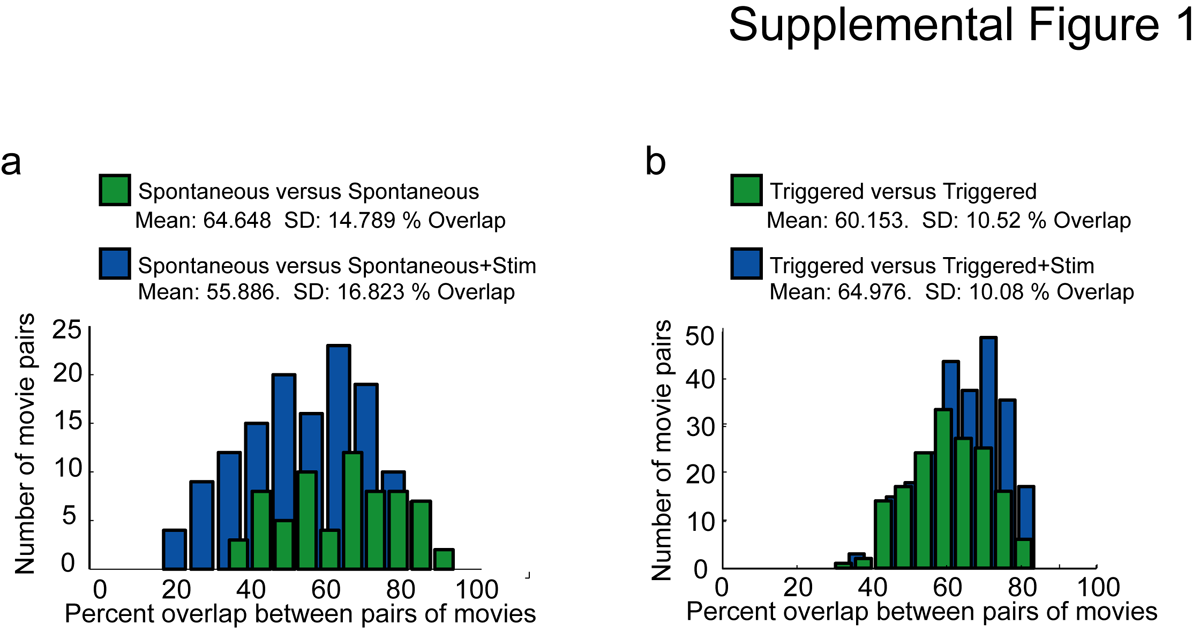

Supplement: Figure S1 — Effect of thalamic inputs on coactivation overlaps, segregated by type of UP state. (a) Overlap of pairs of movies during pairs of spontaneous UP state events (green) and pairs comprised of one spontaneous UP state and one spontaneous UP state with impinging thalamic stimulation (blue). The difference between the means of these distributions was not significant (p>0.10). (b) Same analysis carried out with pairs of movies during pairs of thalamically triggered UP state events (green) and pairs comprised of one thalamically triggered UP state and one thalamically triggered UP state with impinging (additional) thalamic stimulation (blue). The difference between the means of these distributions was not significant (p>0.10). (2.28 MB TIF) [file pone.0003971.s001.tif]

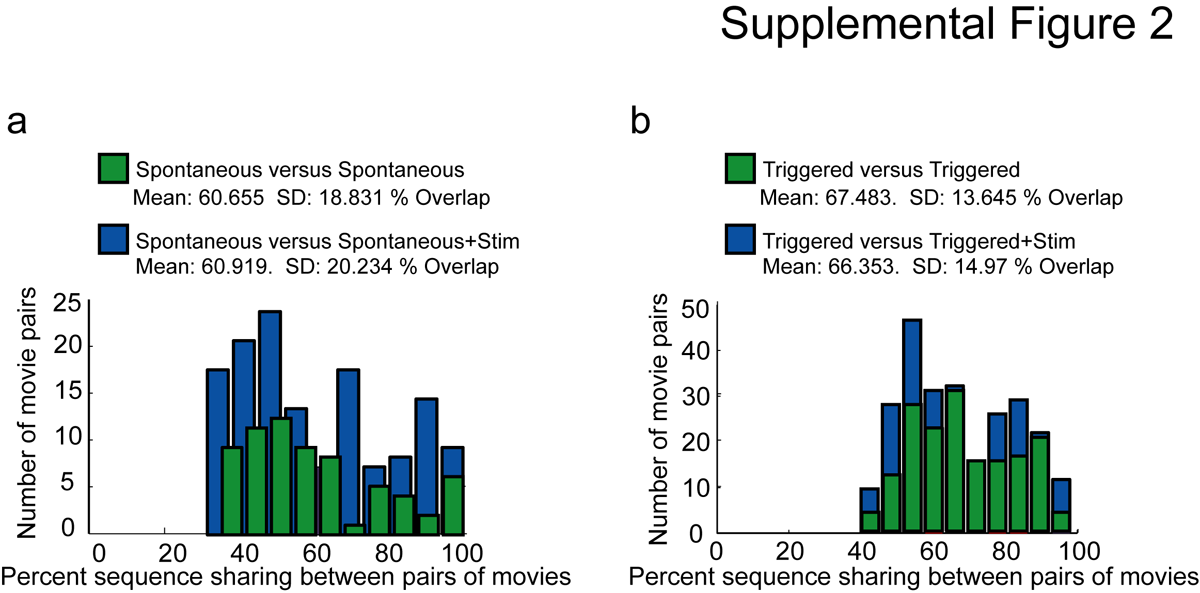

Supplement: Figure S2 — Analysis of effect of thalamic input on temporal sequences overlap segregated by type of UP state. (a) Sequence sharing from pairs of movies during pairs of spontaneous UP state events (green) and pairs comprised of one spontaneous UP state and one spontaneous UP state with impinging thalamic stimulation (blue). The difference between the means of these distributions was not significant (p>0.10). (b) Same analysis carried out with pairs of movies during pairs of thalamically triggered UP state events (green) and pairs comprised of one thalamically triggered UP state and one thalamically triggered UP state with impinging (additional) thalamic stimulation (blue). The difference between the means of these distributions was not significant (p>0.10). (2.15 MB TIF) [file pone.0003971.s002.tif]

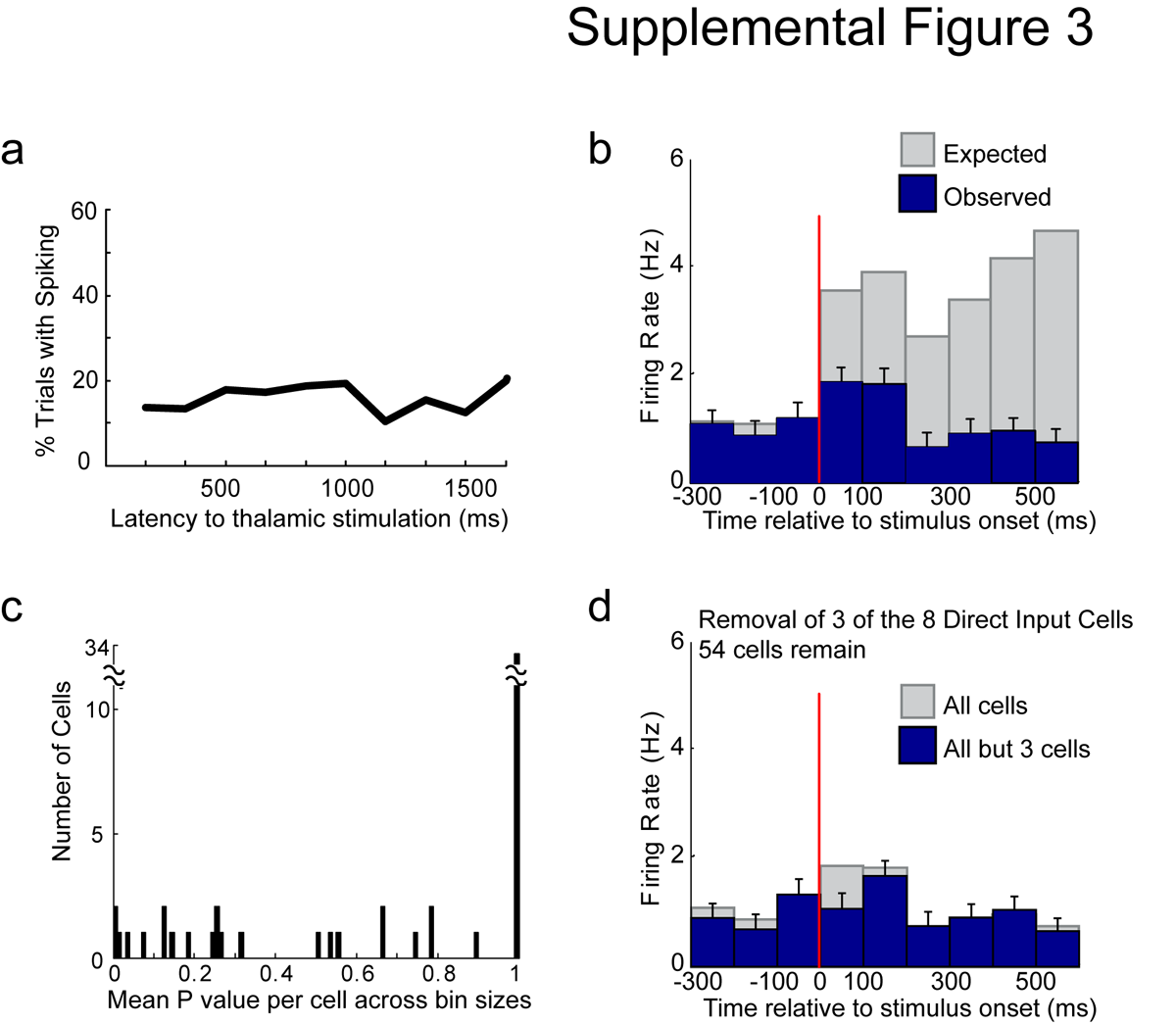

Supplement: Figure S3 — Lack of effect of thalamic stimulation on the temporal activity during ongoing UP states. (a) Plot of percent likelihood of action potential generation in response to thalamic stimulation during ongoing UP states over the duration of the UP state (moving average with 1000 ms bins, 250 ms apart). Time of thalamic stimulation relative to UP state start time does not greatly affect the responsivity of the cortex to thalamic input. Time histograms centered at times of thalamic stimulation in the DOWN state versus the UP state in 57 neurons. (b) Expected versus observed response to stimulation during ongoing UP states) shown in peri-stimulus time histogram (PSTH) format (bin width = 100 ms, stimulation time t = 0 represented as vertical red line). Gray bars show linear summation of firing rate during ongoing UP states with that observed after thalamic stimulation in the DOWN state from all 57 cells examined. Blue bars show observed firing before during and after stimulation during UP states. Following the start of stimulation, there is a small increase in spike rate across all cells, which is significantly greater than baseline under some binning regimes but not others. Also it is significantly less than expected (p<0.05 by bootstrap resampling of first and second bins in observed dataset). (c) Histogram of average p values for test of greater than expected post-stimulus spiking for each cell over analyses using multiple bin sizes. P values calculated by reshuffling stimulation times over the duration of UP states to determine spiking expected by chance. P values for neurons were distributed uniformly, as 34 of 57 neurons showed post-stimulus spiking either less than or equal to that expected from their overall spike rate (p = 1). The remaining 27 cells had p values distributed relatively evenly between 0 and <1. Three neurons demonstrated both a mean p value less than 0.5 but also p values less than 0.5 across each and every bin size. (d) In blue bars is the population [file pone.0003971.s003.tif]

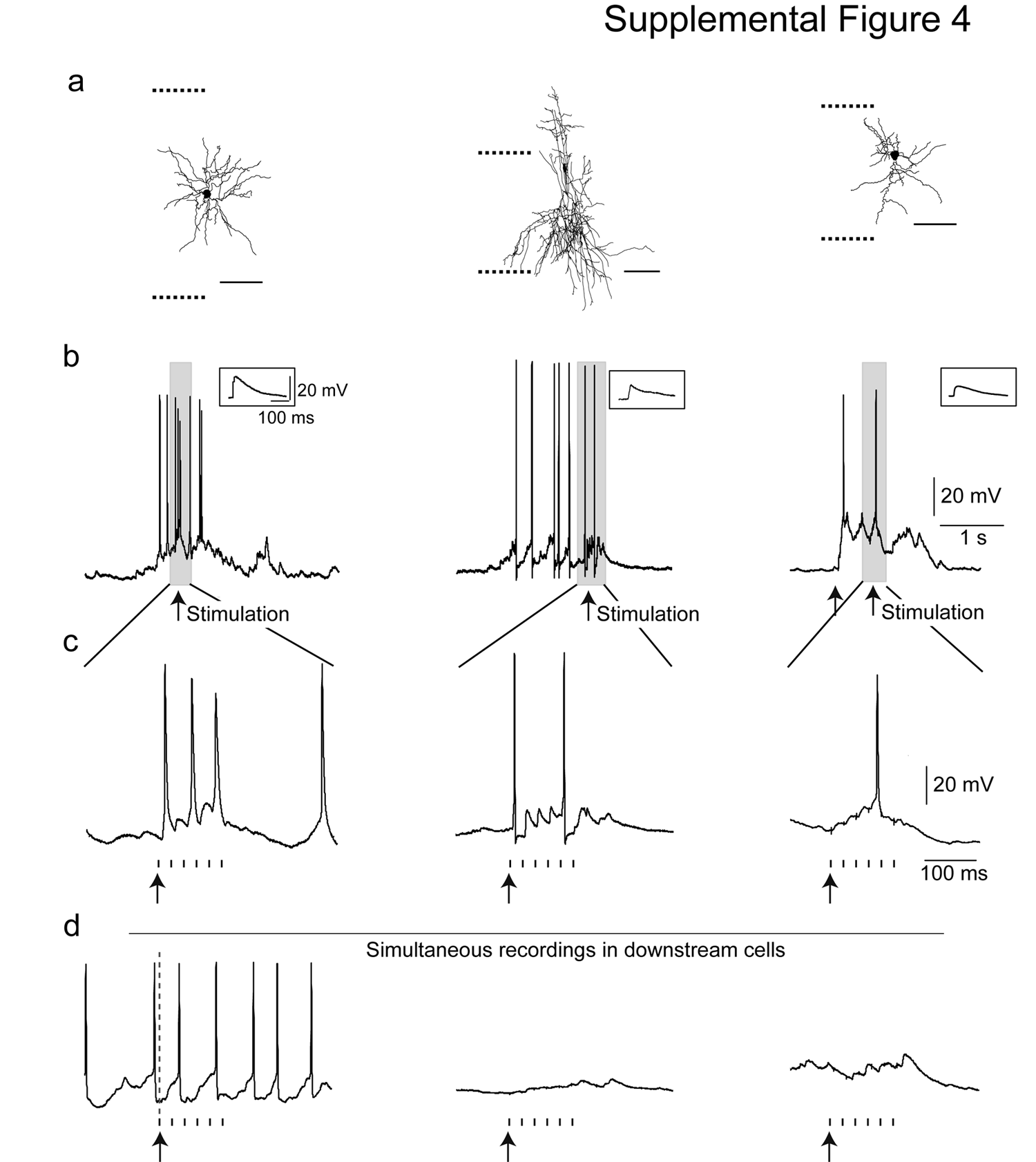

Supplement: Figure S4 — Three direct-input neurons display increased spiking after thalamic stimulation during UP states. Three neurons showing significantly greater than chance spiking after thalamic stimulation during UP states across all time binning strategies. Each neuron is represented in a column; thalamic stimulations were only delivered during spontaneous UP states in the cells in left two columns, while thalamic stimulations were only delivered during ongoing thalamically stimulated UP states in the right column. (a) Reconstructions of each responding neuron. Layer 4 upper and lower boundaries are indicated by dotted lines. Scale bars 100 um. (b) UP states during which thalamus was stimulated, with stimulus times indicated by arrows. Peri-stimulus times are shown in gray boxes and are shown at higher temporal resolution below. (c) Action potentials are clearly triggered during the period of stimulation, but during UP states are restricted to that time. (d) Recordings made simultaneously with those in the third row, but in other neurons, none of which received direct thalamic input. Consistent with our other observations in 46 other neurons not receiving direct thalamic input, even these cells which are recorded simultaneously with consistently responsive up stream cells demonstrate no spiking response following thalamic stimulation. (2.54 MB TIF) [file pone.0003971.s004.tif]

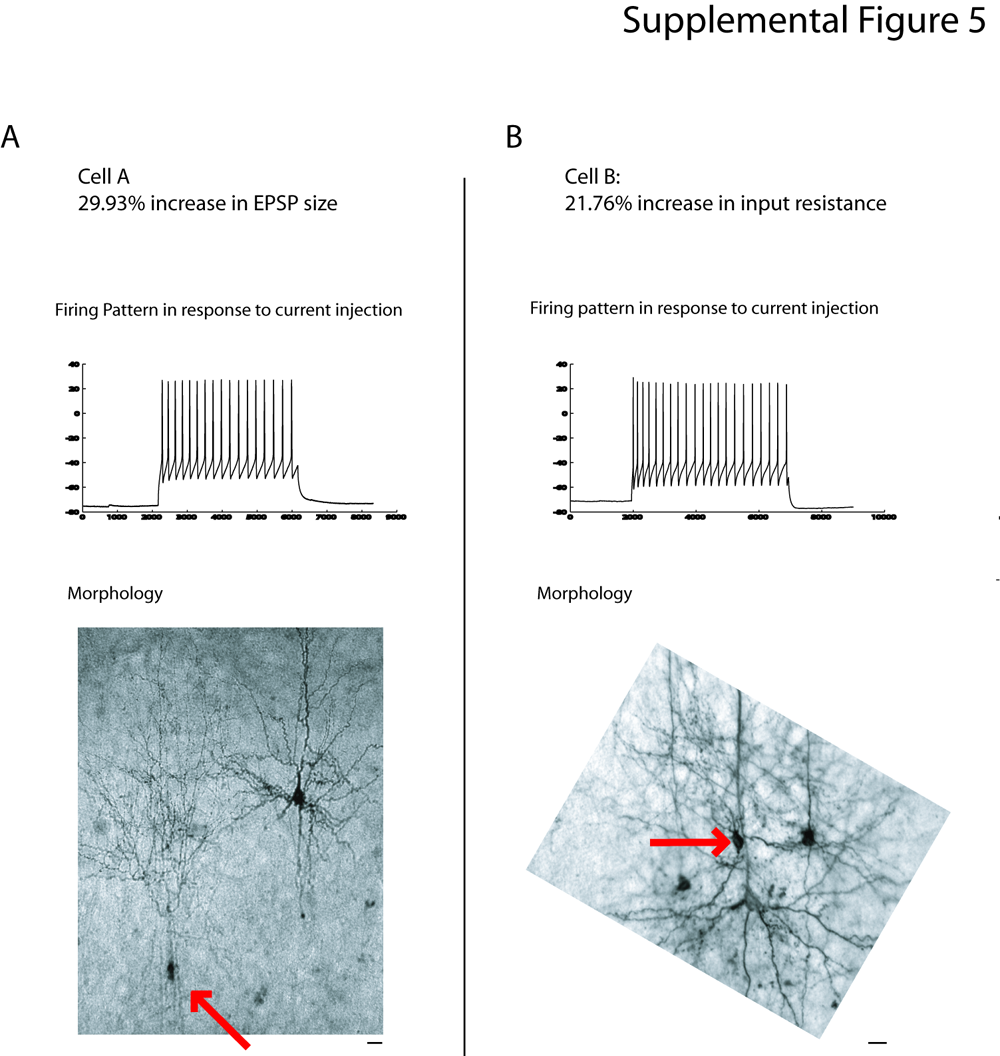

Supplement: Figure S5 — Inhibitory interneurons with apparently increased responsiveness during UP states. Side by side comparison of interneurons, one of which received direct input from thalamus (a) and received 29.9% larger amplitude thalamocortical EPSPs during UP states than during down states and the other (b) demonstrated 21.76% increased input resistance during UP states. Upper panels: Both neurons were members of the interneuron subtype with fast spiking in response to depolarizing current injection and had strikingly similar action potential and after hyperpolarization kinetics. Lower panels: Biocytin fills of both cells; each was recorded simultaneously with other neurons. Interneurons of interest indicated with red arrows. Scale bars 10 µm. (4.24 MB TIF) [file pone.0003971.s005.tif]
